# Prospective analysis of the attendance behaviour of the participants of a facilitated support group for patients after allogeneic hematopoietic cell transplantation

**DOI:** 10.1007/s00520-023-08279-0

**Published:** 2023-12-27

**Authors:** Karsten Geeck, Sebastian Kreil, Michaela Hausmann, Wolf-Karsten Hofmann, Daniela Heidenreich, Stefan A. Klein

**Affiliations:** Department of Hematology and Oncology, University Hospital Mannheim, Heidelberg University, Theodor-Kutzer-Ufer 1-3, D-68167 Mannheim, Germany

**Keywords:** Allogeneic hematopoietic cell transplantation, Support group, Participation behaviour, Participation rates

## Abstract

**Purpose:**

Support groups might help survivors of allogeneic hematopoietic cell transplantations (HCT) to cope with medical, psychological, and social challenges. The aim of this project was (1) to establish a facilitated post-HCT support group and (2) to assess the participation behaviour.

**Methods:**

From 11/2013 until 7/2017, all adult patients who had received a HCT at our centre were invited to participate in a professionally facilitated support group. The format of the group was unstructured without any rules regarding regular attendance. The attendance was prospectively minuted by the facilitator. Reasons for non-attendance were assessed by a survey.

**Results:**

During the observation period, 53 group meetings were scheduled. Nine meetings were cancelled because of low attendance. Altogether 23 different patients (F: *n*=10; M: *n*=13) and 10 spouses (F: *n*=9; M: *n*=1) participated. Median participation was 5 [range 2–11]. With respect to all HCT patients who had the theoretical opportunity to attend, the mean participation rate was 7%. Thirteen patients and four spouses attended more than one meeting. The median count of participations among those participants was 8 [2-32]. The median interval from the first until the last participation was 16 months. The main reason reported for non-participation was the effort to get to the venue of the support group.

**Conclusions:**

To our knowledge, this is the first analysis on the attendance behaviour of the participants of a support group for HCT survivors. The results provide guidance for the organization of future support groups and indicate what participation rates can be expected and how they might be increased.

## Introduction

Support groups have become widespread in very different areas of life and, especially in the medical field, represent a supportive instrument for coping with crisis situations and stressful phases of life [1]. Patients who underwent an allogeneic hematopoietic cell transplantation (HCT) are confronted with medical, psychological, and social challenges [2–5]. One could therefore assume that particularly these patients develop a special need for support and that self-help groups should be of considerable importance. Support groups for patients with solid tumours have become widespread during the last two to three decades [6–8]. Several studies addressed the issue of participation in support groups among patients with solid tumours [8, 9]. On the other hand, little is known about the implementation of support groups for patients after allogeneic HCT. Scientific data on support groups for these patients are scarce. More than 20 years ago, Marks and Benjamin reported on the establishment of a support group for patients after allogeneic HCT [10]. Lounsberry et al. published a feasibility study of a telehealth-delivered psychoeducational support group for patients after allogeneic HCT in 2010 [11]. However, recent data on support groups for patients after allogeneic HCT are not available. Moreover, data on the participation behaviour of members of a post-HCT support group are completely lacking. The aim of this project was (1) to establish a post-HCT support group and (2) to assess the attendance behaviour of its participants. In order to relieve the participants of the burden of organization, the design of a facilitated support group was chosen. A further intention for this design was to ensure a possibly necessary coordination of the contributions.

## Methods

### Patients

All adult patients (≥ 18 years) who had received an allogeneic HCT at the University Hospital Mannheim since the start of its activity in 07/2010 were invited to participate in a professionally facilitated support group from 11/2013 until 7/2017. Twice per year, patients were invited by mail together with a list of the scheduled meetings. In addition, posters announcing the meetings were displayed in the HCT outpatient clinic. Patients were encouraged to bring their spouse.

### Design

For this prospective study, the design of a facilitated support group was chosen. The role of the facilitator was carried out by a physician who is also a catholic theologian. Over the entire period of 4 years, the role of the facilitator was filled by the same person. This facilitator was not part of the HCT team and thus represented a neutral person. His role was to organize the sessions and coordinate the group discussions. He did not participate as a discussant, nor did he influence the content. The meetings were unstructured discussion rounds. Topics were not specified. The meetings did not have an educational character.

During the first year, the group meetings were scheduled every 14 days. The participants decided to reduce the frequency of meetings to once a month starting in 01/2015. Meetings with an expected number of participants of fewer than two persons were not held. Each meeting lasted for 90 min. In order not to interfere with the meetings by negative memories or emotions related to the transplantation or other medical procedures, a seminar room in the library building of Mannheim medical school was chosen as a neutral venue outside the premises of the transplantation programme.

### Analysis of attendance behaviour

The primary objective of the study was to assess the participation behaviour of the attendants of the meetings. For this purpose, it was documented how many patients and spouses attended the meetings. In addition, the gender of the participants was recorded. As part of the data analysis, it was evaluated which and how many meetings the individual participants took part. Attendants who participated at least twice were defined as multiple participants. It was recorded when the first and last attendance took place for each individual participant.

To calculate the participation frequency of multiple participants, the actual number of meetings attended by a particular participant was divided by the number of meetings the participant could have attended from his/her first to his/her last attendance time point.

In addition, the participation rate was measured. For this purpose, it was determined for each individual session how many patients could have participated in an optimal setting. Any patient who was alive and whose transplantation was more than 28 days ago was counted as a potential participant. To calculate the participation rate of attendants, the actual number of participants of an individual meeting was divided by the number of potential participants.

### Survey on non-attendance

Reasons for non-attendance were assessed by a survey among patients who have never attended a meeting of the group. The questionnaire was designed specifically of this study. Patients were asked to complete the questionnaire during the waiting period at a routine visit to our HCT outpatient clinic. For this manuscript, the survey questions were translated from German into English.

### Statistical analysis

Statistical analysis was performed using GraphPad Prism™ (La Jolla, CA). The median duration of attendance of multiple participants was determined using a Kaplan-Meier plot.

## Results

### Meetings

**Fig. 1:**
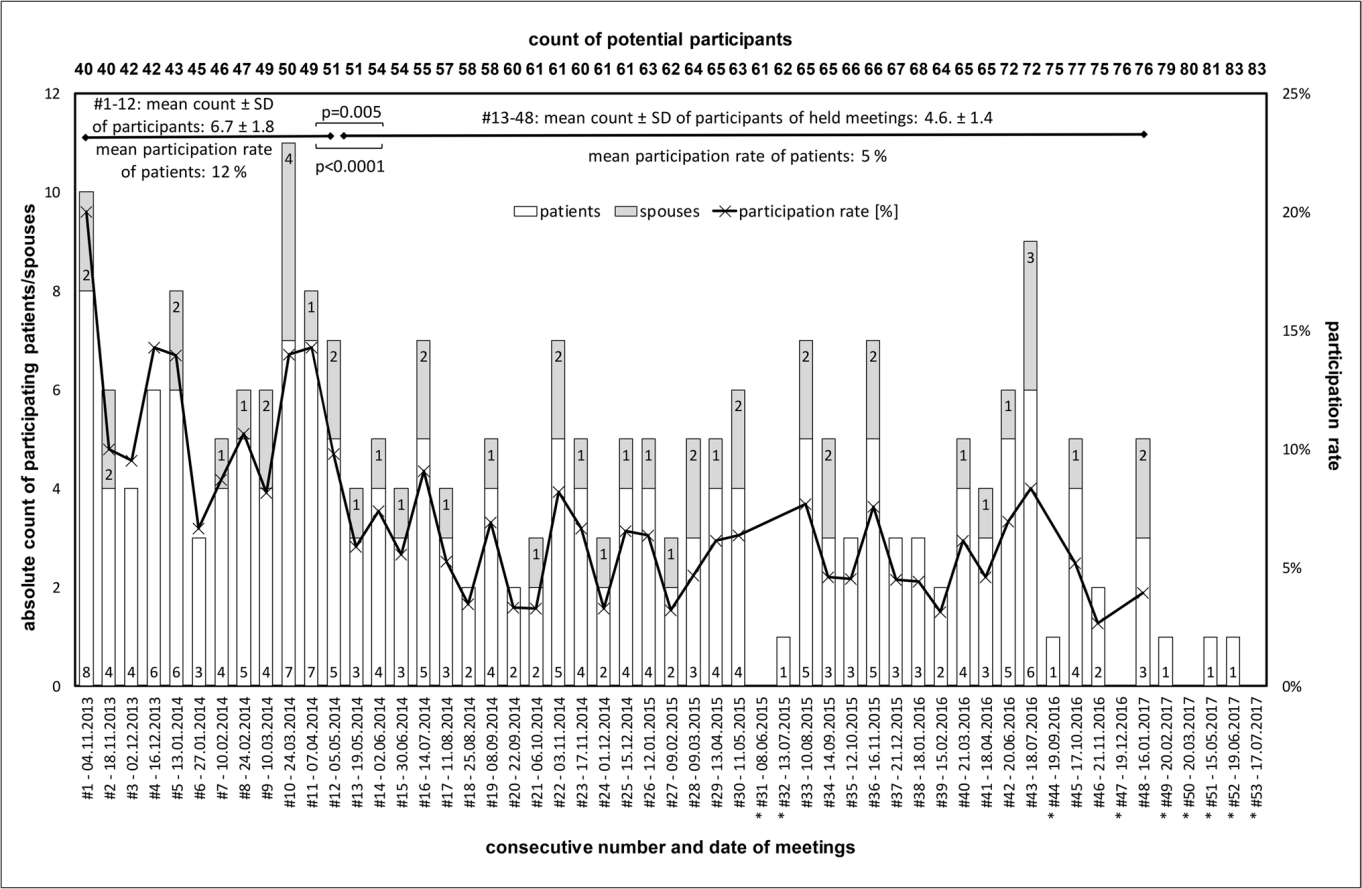
Absolute count of participating patients or spouses (white and grey columns, left y-axis) and participation rate of patients (black graph, right y-axis) for each individual meeting of the group. Meeting date and consecutive meeting number are shown below the x-axis. The number of potential participants is shown at the top. Meetings which were not held because of <2 attendants are marked by an asterisk

During the entire observation period of 45 months a total of 53 group meetings were scheduled. Nine meetings were cancelled because of low attendance (i.e. fewer than two participants, Fig. 1). Of the first 30 meetings planned between 11/2013 and 05/2015, all took place. On the other hand, of the last 10 scheduled meetings (between 09/2016 and 07/2017), seven of them did not take place. For this reason, the meetings were stopped after 07/2017.

### Participants

**Fig. 2:**
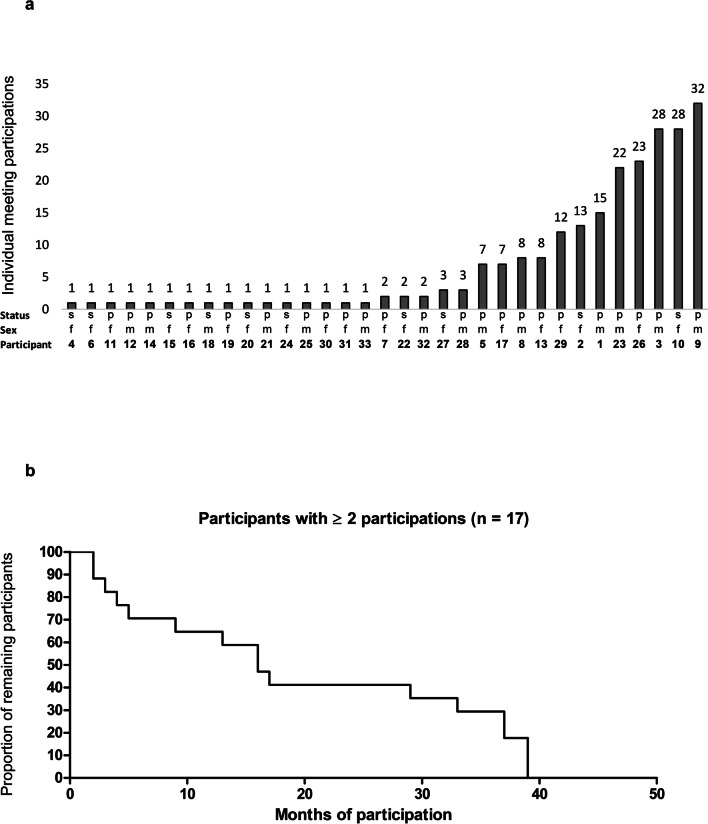
(**a**) Individual number of meeting participations of the participants. Each individual participant is characterized by its participation number, sex [male (m)/female (f)] and the status [patient (p)/spouse (s)]. (**b**) Kaplan-Meier plot of the proportion of remaining participants. Depicted are the data of all multiple participants (≥ 2 participations)

Altogether, a total of 33 different persons attended the group meetings (Table 1): 23 patients (F: *n*=10; M: *n*=13) and 10 spouses (F: *n*=9; M: *n*=1). The number of sessions attended by each participant is presented in Fig. 2a. For each individual participant, gender and status (patient or spouse) are shown, as well as the number of sessions attended. Sixteen participants (10 patients, six spouses; F: *n*=6, M: *n*=10) attended only one single meeting. On the other hand, 17 participants (13 patients, four spouses; F: *n*=9; M: *n*=8) attended multiple (≥2) sessions.

**Table 1:** Distribution of participants

		Female	Male
Participants	33	19	14
Patients	23	10	13
Spouses	10	9	1
Participants (single participation)	16	10	6
Patients	10	5	5
Spouses	6	5	1
Participants (≥ 2 participations)	17	9	8
Patients	13	5	8
Spouses	4	4	0

While men slightly predominated among participating patients, only female spouses participated, with the exception of a single husband. This significant distribution difference could be observed within the entire cohort, among single participants as well as among multiple participants, respectively (Table 1).

The median number of participants in the meetings that actually took place was 5 (range 2–11), i.e. four patients (2–8) and one spouse (1–4), respectively. The median count of participations among those who attended ≥2 meetings was 8 (2–32, Table 2). With regard to the number of participations, there was no significant difference in terms of gender or between patients and spouses.

**Table 2:** Participation frequency

Consecutive no. of participants	Sex (f/m)	Patient/spouse (p/s)	Opportunities to participate*	Actual participations^#^ (%)	
7	f	p	3	2	(67)
13	f	p	15	8	(53)
17	f	p	15	7	(47)
26	f	p	43	23	(53)
29	f	p	27	12	(44)
2	f	s	44	13	(30)
10	f	s	48	28	(58)
22	f	s	9	2	(22)
27	f	s	4	3	(75)
1	m	p	48	15	(31)
3	m	p	46	28	(61)
5	m	p	23	7	(30)
8	m	p	14	8	(57)
9	m	p	48	32	(67)
23	m	p	40	22	(55)
28	m	p	4	3	(75)
32	m	p	2	2	(100)
		**∑**	**433**	**215**	**(50)**
Mean	**f/m: 10.9/14.6 ns**	**p/s: 13.0/11.5 ns**		**12.6**	
Median	**f/m: 8.0/11.5 ns**	**p/s: 8.0/8.0 ns**		**8.0**	

*Count of opportunities to participate counted from the first individual participation of a certain participant until his last possible participation

^#^Count of actual participations of a certain participant

### Participation frequency

The total number of participations of all multiple participants was 215, calculated from the respective first to the last personal attendance of multiple participants. The total of all opportunities to participate was 433. Excluding patient #32, who attended two out of two possible meetings, the individual participation frequency per patient ranged between 22 and 75%. In absolute terms, participant #9 attended the most meetings with 32 participations out of 48 possible opportunities. The average participation frequency of multiple participants was 50% (Table 2). The median duration of attendance of the multiple participants was 16 months (Fig. 2b).

### Participation rate

The participation rate in relation to all allogeneic HCT patients at our institution was calculated as the quotient of the number of patients who attended a specific meeting and the allogeneic HCT patients who theoretically could have attended that meeting. Overall, the average participation rate was 7% (range 3–20%, Fig. 1).

Both the mean number of participants and the participation rate were significantly higher during the first 6 months of the meetings (meetings #1–12) than beyond meeting 12 (mean number of participants 6.7 versus 4.6 (*p*=0.005), mean participation rate 12% versus 5% (*p*<0.0001)).

### Survey on reasons for non-attendance

**Fig. 3:**
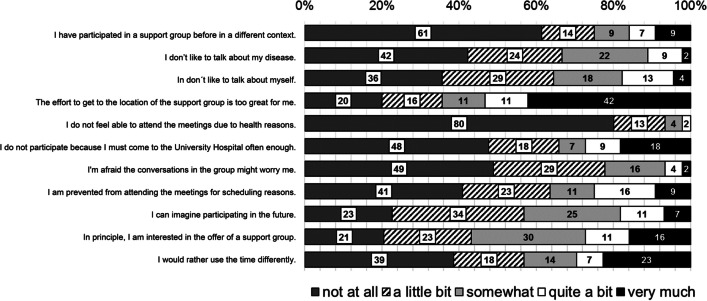
Results of the survey on reasons for non-participation in the support group. The results are presented in per cent

Forty-seven patients were asked to complete the questionnaire on reasons for non-attendance. Forty-five questionnaires (96%) were returned completed and analysed thereafter. Two patients were excluded from the analysis: One patient returned a blank questionnaire. The second patient met an exclusion criterion for the survey because he had attended a meeting of the support group. The results of the survey are shown in Fig. 3.

Most respondents chose “The effort to get to the location of the support group is too great for me” (42% very much, 11% quite a bit) as a reason for not participating. No other statement met with agreement from the majority of survey participants. With an incidence of 23% (very much) and 7% (quite a bit), respectively, the statement “I would rather use the time differently” was cited as the second most common reason. Indicating the limited enthusiasm for the meetings, only a minority agreed with the statement “In principle, I am interested in the offer of a support group” (16% very much, 11% quite a bit). The most frequently negated statement was “I do not feel able to attend the meetings due to health reasons” (not at all, 80%). Psychological reasons such as fear that the conversations in the group might worry or that one does not like to talk about oneself or the disease played almost no role for non-participating (2%, 4%, and 2% very much; 4%, 13%, and 9% quite a bit, respectively).

## Discussion

The experience of an allogeneic HCT has a profound impact on HCT recipients [3]. HCT survivors are challenged by medical, psychological, and social complications [3, 12–24]. For patients with solid tumours or chronic diseases, support groups have emerged over the past three decades as a successful tool for coping with the evolving stress associated with the disease and/or diagnostic or therapeutic procedures [25–28]. However, scientific data on support groups for HCT survivors are scarce [10, 11, 29–32]. With this prospective study, we report on establishing a support group for patients after allogeneic HCT. As far as we know, this is the first analysis on the participation behaviour of members of a support group for HCT survivors.

Forty-four support group meetings were attended by cumulative 33 different participants over a period of 45 months. Almost one-third of the participants were spouses of the patients. While the sex ratio among patients was balanced and roughly corresponded to the sex ratio of transplanted patients, female participants significantly predominated among spouses. Both the numerical proportion of spouses and the predominance of females are in line with the results of the SHILD study on participation behaviour in different types of self-help groups of patients with chronic diseases or cancer [33, 34].

Of note, nearly half of the participants attended a meeting only once. On the other hand, multiple participants attended support group meetings for a median of 16 months. This attendance behaviour suggests that at their first meeting, participants made a principal decision either not to attend again or to attend for a longer period. The reasons for this decision can only be speculated. Trojan et al. showed that the majority of participants in self-help groups previously had little knowledge about these groups [34]. Perhaps the offer of the facilitated group described here did not meet the expectations of some of the participants. Possibly, particularly educational elements were missed, which were not part of this facilitated group concept. Marks and Benjamin reported in 2000 on the request for disease-related information in a self-help group for patients after HCT [10].

Compared with self-help groups for other diseases, a median duration of participation of 16 months appears to be short [7, 33, 34]. However, it has to be considered that allogeneic HCT is a curative therapy. In many survivors, the suffering pressure decreases with time. Boers et al. showed that after 3 years post HCT quality of life was considered good by most patients [5, 16].

The average participation rate was 7%, i.e. the average of the number of participating patients per meeting in relation to the number of potentially available patients on the respective meeting day. This rate is lower than the published rates for self-help groups for patients with chronic diseases or cancer with participation frequencies of 15–33% [9, 34, 35]. However, these analyses were rarely based on a day-by-day consideration as in this study. Data on the participation rates of support groups for HCT survivors are not available. The only comparator in the field of HCT is a feasibility study of a telehealth-delivered psychoeducational support group [11]. In this, the authors report a participation rate of 11%. However, these data are also not based on a day-by-day observation. In contrast to the self-help group presented here, which was planned as a long-term offer without a fixed thematic specification, the approach of Lounsberry et al. was focused on psychoeducation with a pre-defined agenda and only six scheduled meetings.

Of note, the participation rate was significantly higher during the first 6 months of the group meetings than in the following 3 years. Moreover, of the last 10 scheduled meetings, seven were cancelled due to low attendance. Finally, the low participation was the main reason to terminate the offer of the support group. The reduced participation rate from the second to the fourth years can possibly be attributed to the fact that the offer of the support group started more than 3 years after the beginning of the transplantation activity of our centre. From the first group meeting on, there has been a number of 40 potential participants plus spouses, already. The initial participants started to form a kind of a core group during the first meetings. After the size of this core had decreased significantly, the new potential participants of about 20 patients per year were not sufficient in terms of numbers to keep the level of participants at a stable level over a longer period of time.

Since only a minority of potential participants attended the meetings, a survey on the reasons for non-attendance was performed among those who never participated in a meeting of the support group. The response rate to the questionnaires was 96%. This is probably due to the opportunity to answer the survey during the waiting period at routine visits at our HCT outpatient clinic. The survey showed that about half of the non-participants were in principle interested in participating in a support group. Neither physical nor psychological reasons were given as decisive for non-participation. In contrast to studies on reasons for non-participation in support groups among patients with solid tumours, the lack of awareness of the availability of a support group did not play a role in our cohort of non-participants [36–38]. In our survey, the most common reason selected for non-participation was the effort required to get to the location of the meetings. These data provide indications how support groups could be organized to motivate more patients after HCT to participate. Since the additional distance to the meetings was seen as the main problem, group meetings during the waiting time in the outpatient clinic or online meetings, for example, could be alternative offers. Moreover, it should be discussed whether self-help groups can be better integrated into the overall concept of supportive measures after allogeneic HCT. This would offer the possibility to integrate the offer of the group meetings into the routine care and at the same time to lower the inhibition to participate. Another way to increase patients’ interest in participating in a support group was recently presented by Fox et al. for adolescents and young adults [39]. The authors described how online surveys were used to find out the needs of the participants and how the group meetings were modified according to the responses. However, this approach would not be compatible with the unstructured discussion rounds without an educational character of the support group of this study.

The main limitation of this analysis is the relatively small sample size. Additionally, it is worth discussing whether the design as a facilitated support group may have influenced participation behaviour. However, it seems necessary to relieve the overall heavily burdened patients after HCT of the additional load of organizing a support group. In addition, it should be discussed whether the results of this study can be transferred to online concepts for support groups. Possibly, the participation rate would be significantly higher for online offers.

In summary, this study provides insights into the participation behaviour of patients and their spouses in a support group after HCT. The results provide guidance for the organization of future support groups and indicate what participation rates can be expected and how they might be increased.

## Data Availability

This declaration is not applicable.
